# Administration of a TLR9 Inhibitor Attenuates the Development and Progression of Heart Failure in Mice

**DOI:** 10.1016/j.jacbts.2019.01.002

**Published:** 2019-05-22

**Authors:** Hiromichi Ueda, Osamu Yamaguchi, Manabu Taneike, Yasuhiro Akazawa, Haruko Wada-Kobayashi, Ryuta Sugihara, Hiroki Yorifuji, Hiroyuki Nakayama, Shigemiki Omiya, Tomokazu Murakawa, Yasushi Sakata, Kinya Otsu

**Affiliations:** a Department of Cardiovascular Medicine, Graduate School of Medicine, Osaka University, Suita, Osaka, Japan; b Laboratory of Clinical Science and Biomedicine, Graduate School of Pharmaceutical Sciences, Osaka University, Suita, Osaka, Japan; c School of Cardiovascular Medicine and Sciences, King’s College London British Heart Foundation Centre of Excellence, London, United Kingdom

**Keywords:** heart failure, mitochondria, pressure overload, Toll-like receptor 9, CCCP, carbonyl cyanide *m*-chlorophenyl hydrazine, CpG, cytidine-phosphate-guanosine, CpG ODN, unmethylated cytidine-phosphate-guanosine containing oligodeoxynucleotide, DNA, deoxyribonucleic acid, E6446, (6-[3-(pyrrolidin-1-yl)propoxy)-2-(4-(3-(pyrrolidin-1-yl)propoxy)phenyl]benzo[d]oxazole), EdU, 5-ethynyl-2′-deoxyuridine, IL, interleukin, IVSd, end-diastolic interventricular septal wall thickness, LAMP, lysosome-associated membrane protein, LC, microtubule-associated protein light chain, LPS, lipopolysaccharide, LV, left ventricular, mRNA, messenger ribonucleic acid, TAC, transverse aortic constriction, TLR, Toll-like receptor, TNF, tumor necrosis factor

## Abstract

Mitochondrial deoxyribonucleic acid, containing the unmethylated cytidine-phosphate-guanosine motif, stimulates Toll-like receptor 9 to induce inflammation and heart failure. A small chemical, E6446 [(6-[3-(pyrrolidin-1-yl)propoxy)-2-(4-(3-(pyrrolidin-1-yl)propoxy)phenyl]benzo[d]oxazole)], is a specific Toll-like receptor 9 inhibitor in cardiomyocytes. In this study, we showed that E6446 exerts beneficial effects for the prevention and treatment of pressure overload–induced heart failure in mice. When administered before the operation and chronically thereafter, E6446 prevented the development of left ventricular dilatation as well as cardiac dysfunction, fibrosis, and inflammation. Furthermore, when administered after the manifestation of cardiac dysfunction, E6446 slowed progression of cardiac remodeling. Thus, the inhibitor may be a novel therapeutic agent for treating patients with heart failure.

## Highlights

• Under pressure overload, mitochondrial deoxyribonucleic acid containing the unmethylated cytidine-phosphate-guanosine motif is accumulated in cardiomyocytes and stimulates Toll-like receptor 9, resulting in inflammation and heart failure.
• Treatment with E6446, (6-[3-(pyrrolidin-1-yl)propoxy)-2-(4-(3-(pyrrolidin-1-yl)propoxy)phenyl]benzo[d]oxazole), a specific Toll-like receptor 9 inhibitor, prevented the development and slowed the progression of left ventricular dilatation and cardiac dysfunction in mice after pressure overload.
• E6446 attenuated the inflammatory responses in the pressure-overloaded mouse heart, even though the accumulation of mitochondrial deoxyribonucleic acid in cardiomyocytes was observed.
• E6446 could be a new therapeutic agent against heart failure.

## Visual Abstract

**Figure:**
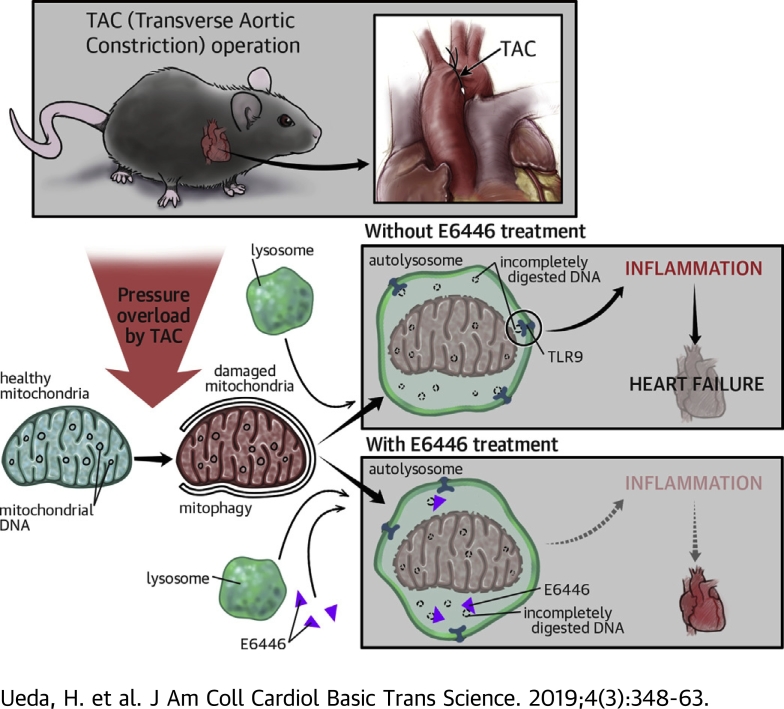

Heart failure is a complex disease associated with high levels of morbidity and mortality and marked reductions in quality of life. Previous extensive studies on heart failure have reported an important role for proinflammatory cytokines in its pathogenesis (1). Circulating levels of proinflammatory cytokines, including tumor necrosis factor (TNF)-α, are related to the severity and prognosis of the disease. However, the targeted anti-TNF-α approaches were neutral with respect to the primary endpoints of the trial or resulted in worsening heart failure or death 2, 3. In addition to TNF-α, the pro-inflammatory cytokines that are elaborated in heart failure include other members of the TNF superfamily, members of the interleukin-1 family, and interleukin (IL)-6 (1). Recognizing the molecular mechanism underlying the developing inflammation in heart failure is essential for developing strategies to control disease progression, including therapeutic drugs.

Mitochondrial deoxyribonucleic acid (DNA) contains the unmethylated cytidine-phosphate-guanosine (CpG) motif, which stimulates Toll-like receptor (TLR) 9 to induce inflammation 4, 5. Mitochondria damaged by external hemodynamic stress are degraded by the autophagy/lysosome system in cardiomyocytes (6). Insufficient degradation of mitochondrial DNA mediated through autophagy in pressure-overloaded mouse hearts leads to its binding to TLR9 to induce inflammation and heart failure (7). In failing mouse hearts, mitochondrial DNA is located in autolysosomes. Furthermore, TLR9 ablation in pressure-overloaded mice attenuated the development of inflammation and heart failure. Thus, interference with TLR9 function by small molecules is likely to produce a better clinical outcome by preventing its aberrant inflammatory responses.

E6446 (6-[3-(pyrrolidin-1-yl)propoxy)-2-(4-(3-(pyrrolidin-1-yl)propoxy)phenyl]benzo[d]oxazole), is a synthetic antagonist of nucleic acid–sensing TLRs and is orally bioactive 8, 9. In vitro, E6446 specifically inhibits the activation of TLR9 (8). Others have reported that the compound inhibits TLR9 but also TLR7 in a ligand-dependent manner (9). When E6446 is administered to mice, it suppresses inflammatory responses to challenge doses of unmethylated CpG containing oligodeoxynucleotide (CpG ODN) 8, 9. When E6446 is administered chronically in mouse cerebral malaria and spontaneous lupus models, the compound inhibits cytokine production with prevention of signs of cerebral malaria and circulating antinuclear antibodies, respectively.

In the present study, the efficacy of oral treatment with E6446 was evaluated on mouse pressure overload–induced heart failure models. Our results indicate that E6446 exerts beneficial effects for the prevention and treatment of heart failure in mice and might be a novel therapeutic agent for treating patients with heart failure.

## Methods

### Cell culture

Adult cardiomyocytes were isolated from 10- to 12-week-old C57BL/6J male mice (CLEA Japan, Inc., Tokyo, Japan) by using a Langendorff system and were then cultured (7).

### Ribonucleic acid analysis

Total ribonucleic acid (RNA) was extracted from the left ventricle or cultured cardiomyocytes by using the TRIzol reagent (Thermo Fisher Scientific, Waltham, Massachusetts) and reverse transcribed by using TaqMan Reverse Transcription Reagents (Thermo Fisher Scientific) (7). Real-time quantitative polymerase chain reaction was performed by using the Platinum Quantitative PCR SuperMix-UDG (Thermo Fisher Scientific). Relative levels of gene expression were normalized to the *Gapdh* messenger RNA (mRNA) expression. The primers (Thermo Fisher Scientific: Assay identity) used were as follows: *Nppa*, Mm01255747_g1; *Nppb*, Mm00435304_g1; *Col1a2*, Mm01165107_m1; *Col3a1*, Mm00802331_m1; *Gapdh*, 4352339E; *Il6*, Mm99999064_m1; *Il1b*, Mm01336189_m1; and *Tnfa*, Mm00443260_g1.

### Immunofluorescence microscopy

Adult mouse cardiomyocytes on laminin-coated glass-based dishes (IWAKI Cell Biology, Bio-REV Pte. Ltd., Singapore) were incubated with 100 nmol/l carbonyl cyanide *m*-chlorophenyl hydrazine (CCCP) for 6 h. To estimate mitochondrial membrane potential, the cells were treated with 10 nmol/l of tetramethylrhodamine ethyl ester (Molecular Probes, Eugene, Oregon) for 30 min. To visualize DNA and autophagosomes, the cells were incubated in three-dimensional gel with Cellmatrix I-A (Nitta Gelatin Inc., Osaka, Japan) and fixed with methanol at −30°C for 15 min. The cells were incubated with anti–microtubule-associated protein light chain (LC) 3B antibody (Cell Signaling Technology, Danvers, Massachusetts) overnight at 4°C, followed by staining with anti-rabbit Alexa 568 secondary antibody (Abcam, Cambridge, United Kingdom) overnight. The cells were incubated with 100-fold diluted PicoGreen (Thermo Fisher Scientific) for 30 min before confocal microscopic analysis using an FV-1000D microscope (Olympus, Tokyo, Japan) (7).

### Animal study

The investigation conforms to the Position of the American Heart Association on Research Animal Use adopted by the American Heart Association on November 11, 1984. All in vivo and in vitro experimental protocols were conducted under the supervision of the Animal Research Committee of Osaka University and in accordance with the Guidelines for Animal Experiments of Osaka University and the Japanese Animal Protection and Management Law.

The 10- to 12-week-old male C57BL/6J mice were subjected to transverse aortic constriction (TAC) surgery (10). Sham-operated animals underwent the same operation without aortic constriction. Blood pressure was measured noninvasively on mice anesthetized with 2.5% tribromoethanol by using a pressure monitor (Model MK-2000, Muromachi Kikai Co., Ltd., Tokyo, Japan). The pressure gradient across TAC was estimated by the difference in blood pressure between both arms by using a pressure monitor. Ultrasonography (Sonos 5500, equipped with a 15-MHz linear probe, Philips Medical Systems, Cambridge, Massachusetts) was used for assessing left ventricular (LV) size and function on conscious mice.

### Drug and treatment

E6446 (Eisai, Inc., Andover, Massachusetts) was dissolved in dimethyl sulfoxide (final 0.04% v/v) for experiments. Isolated cardiomyocytes were pretreated with E6446 (0 to 10 μmol/l) for 1 h, followed by treatment with 1 μg/ml of lipopolysaccharide (LPS) (FUJIFILM WAKO Pure Chemical Co., Osaka, Japan), 2 mmol/l of loxoribine (InvivoGen, San Diego, California), or 5 μmol/l of type B CpG ODN (ODN1668, InvivoGen) for 6 h. To examine the effect of E6446 on the level of cytokine mRNAs, cardiomyocytes were pretreated with 10 μmol/l of E6446 for 1 h, followed by treatment with 100 nmol/l of CCCP for 6 h.

For the in vivo ODN1668 challenge experiments with E6446, the mice were orally administered E6446 at a dose of 1.5 mg/200 μl per mouse using animal feeding needles (Natsume Seisakusho Co., Ltd., Tokyo, Japan). One, two, or 3 days later, the mice were injected intraperitoneally with 60 μg/mouse of ODN1668 2 h before sacrifice, following intraperitoneal injection of 20 mg/mouse of D-galactosamine (MilliporeSigma, Burlington, Massachusetts) for 30 min. In the prevention study, E6446 administration was started 2 days before the operation. Mice were administered E6446 orally at a dose of 1.5 mg/mouse or its vehicle (saline) as a control every 2 days. In the treatment study, 30 mice were subjected to TAC operation for 2 weeks. Ten mice with fractional shortening >50% were excluded from the study. The remaining 20 mice were randomized to the saline- and E6446-treated groups and then orally administered saline or E6446 (1.5 mg/mouse) every 2 days (Table 1).

**Table 1:** Echocardiographic Parameters of All Mice Subjected to TAC Operation for 2 Weeks

	Baseline (n = 30)	TAC for 2 Weeks (n = 30)
LVDd, mm	2.31 ± 0.02	2.77 ± 0.03∗
LVDs, mm	0.88 ± 0.01	1.45 ± 0.03∗
LVFS, %	62.0 ± 0.40	47.8 ± 0.71∗
IVSd, mm	0.92 ± 0.00	1.04 ± 0.01∗
LVPWd, mm	0.86 ± 0.01	1.02 ± 0.01∗
Heart rate, beats/min	696 ± 3.4	685 ± 4.3
LV mass, mg	58.7 ± 0.7	96.6 ± 2.1∗

The echocardiographic parameters of the mice were obtained 2 weeks after the operation. Values are mean ± SE.

IVSd = end-diastolic interventricular septal wall thickness; LV = left ventricular; LVDd = end-diastolic left ventricular internal dimension; LVDs = end-systolic left ventricular internal dimension; LVFS = left ventricular fractional shortening; LVPWd = end-diastolic left ventricular posterior wall thickness; TAC = transverse aortic constriction.

∗ p < 0.05 vs. baseline.

A blood sample was taken from the right ventricle. IL-6, IL-1B, and TNF-α serum levels were measured with an enzyme-linked immunoadsorbent assay kit (Thermo Fisher Scientific for IL-6 and IL-1B, R and D Systems [Minneapolis, Minnesota] for TNF-α).

### Histological analysis

Heart samples were fixed in buffered 4% paraformaldehyde solution and embedded in paraffin (10). Fibrosis fraction was quantified by using ImageJ software (National Institutes of Health, Bethesda, Maryland) and expressed as a proportion of the ventricles. For immunohistochemical analysis, frozen heart sections 5-μm thick were fixed in buffered paraformaldehyde and stained with anti-mouse CD45 (R and D Systems), CD68 (Bio-Rad, Hercules, California), Ly6G/C (BD Pharmingen, BD Biosciences, San Jose, California), CD3 (Abcam), and lysosome-associated membrane protein (LAMP) 2a (Thermo Fisher Scientific) antibodies. To measure the cardiomyocyte cross-sectional area, the frozen heart sections were stained with WGA-Alexa 555 (Thermo Fisher Scientific) for 1 h. The cardiomyocyte cross-sectional area was measured by tracing the outline of >100 cardiomyocytes in each section by using ImageJ software. For detection of mitochondrial DNA, mice were intraperitoneally injected with 5 mg of 5-ethynyl-2′-deoxyuridine (EdU) (Invitrogen) 1 day before sacrifice. EdU was detected by using a Click-iT EdU Alexa Fluor 488 Imaging Kit (Thermo Fisher Scientific) (7). EdU- and LAMP2a double-positive deposits were counted in 5 different areas per section in each mouse.

### Statistical analysis

Results are shown as mean ± SE. GraphPad Prism version 7.04 (GraphPad Software, La Jolla, California) was used for statistical analysis. A Student’s *t*-test was used for a 2-group comparison; a 1-way analysis of variance followed by Tukey’s post hoc test or 2-way repeated measure analysis of variance followed by Tukey’s post hoc test were used for multiple comparisons. Significant differences were defined as p < 0.05.

## Results

### Effect of E6446 on cytokine mRNA production in isolated cardiomyocytes

**Figure 1:**
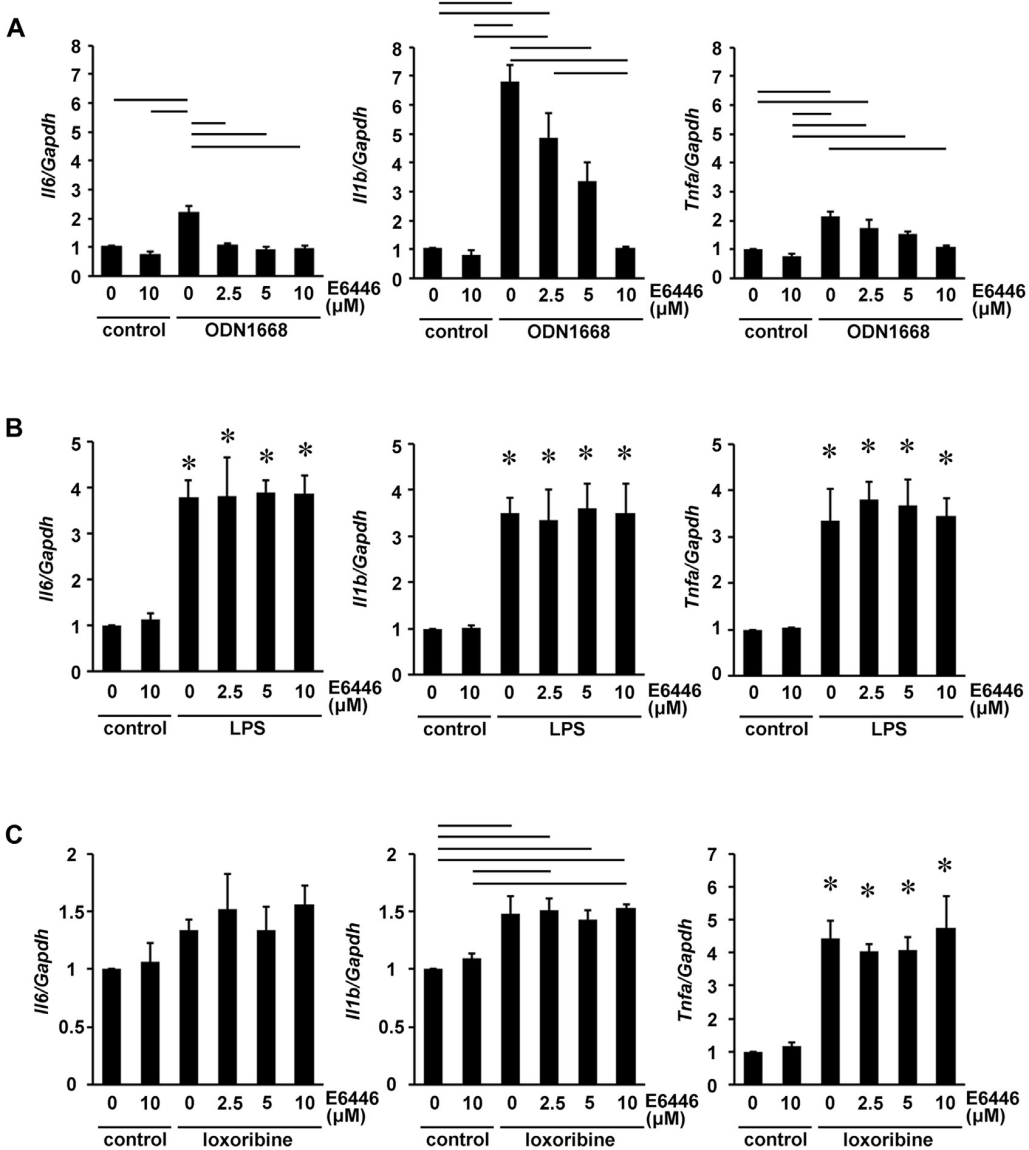
Selectivity of E6446 The expression level of cytokine messenger ribonucleic acid in cardiomyocytes with increasing concentrations of E6446, (6-[3-(pyrrolidin-1-yl)propoxy)-2-(4-(3-(pyrrolidin-1-yl)propoxy)phenyl]benzo[d]oxazole) (n = 3). Cardiomyocytes were treated with 0 to 10 μmol/l of E6446 for 1 h, followed by **(A)** 5 μmol/l ODN1668, **(B)** 1 μg/ml of lipopolysaccharide (LPS), or **(C)** 2 mmol/l of loxoribine for 6 h. Control groups were treated with vehicle. Data were normalized to the content of *Gapdh* messenger ribonucleic acid. Values are mean ± SE. **Bars in graphs** indicate p < 0.05. *p < 0.05 versus both control groups.

Isolated cardiomyocytes were stimulated by a TLR9 ligand (e.g., ODN1668), a TLR4 ligand (e.g., LPS), or a TLR7 ligand (e.g., loxoribine) in the presence of increasing concentrations of E6446 11, 12. ODN1668 significantly increased the expression levels of *Il6*, IL-1B (*Il1b*), and *Tnfa* mRNAs (Figure 1A). Incubation of cardiomyocytes with E6446 significantly reduced the induction of *Il6*, *Il1b,* and *Tnfa* mRNAs in response to ODN1668. LPS significantly increased the expression levels of *Il6*, *Il1b,* and *Tnfa* mRNAs, whereas loxoribine significantly increased the expression levels of *Il1b* and *Tnfa* mRNAs but not *Il6* mRNA (Figures 1B and 1C). E6446 had no effect on the induction of the cytokine mRNAs induced by LPS or loxoribine.

**Figure 2:**
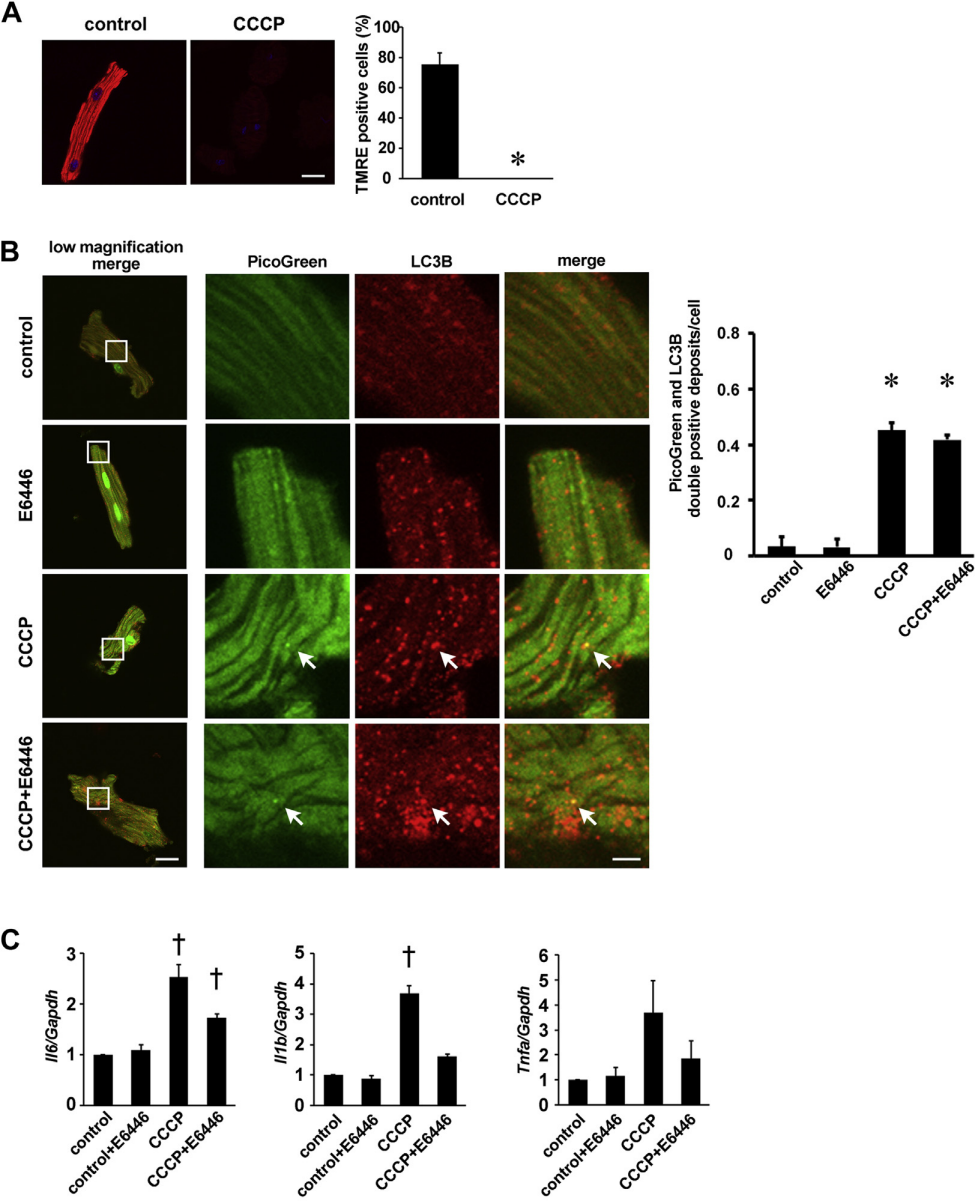
Effect of E6446 on Mitochondrial Damage–Induced Cytokine Messenger Ribonucleic Acid Production in Isolated Adult Mouse Cardiomyocytes **(A)** Disruption of mitochondrial membrane potential by carbonyl cyanide *m*-chlorophenyl hydrazine (CCCP). Isolated cardiomyocytes were incubated with 100 nmol/l CCCP for 6 h and stained with tetramethylrhodamine ethyl ester (TMRE). Scale bar, 20 μm. The **right graph** shows the proportion of TMRE-positive cells. **(B)** Double staining of E6446-treated cardiomyocytes stimulated with CCCP using PicoGreen **(green)** and anti–microtubule-associated protein light chain (LC) 3B antibody **(red)**. Low-magnified images are shown in the **left panels**. Scale bar, 20 μm. Higher magnified images of the squared areas are shown in the **right panels**. Scale bar, 5 μm. **Arrows** indicate PicoGreen and LC3B merged deposits. The **right graph** shows the number of PicoGreen and LC3B double-positive deposits per cell. Ten or more cells were analyzed for each experiment. **(C)** Inhibition of cytokine messenger ribonucleic acid expression in CCCP-stimulated cardiomyocytes by E6446. Cardiomyocytes were treated with 10 μmol/l E6446 for 1 h, followed by 100 nmol/l CCCP for 6 h. Values are mean ± SE (n = 3). *p < 0.05 versus corresponding controls. †p < 0.05 versus all other groups. Abbreviation as in Figure 1.

Incubation of cardiomyocytes with CCCP diminished mitochondrial membrane potential (Figure 2A). Cardiomyocytes were stained with PicoGreen, a highly sensitive marker for DNA, and anti-LC3B antibody, a marker for autophagosomes. CCCP increased the number of PicoGreen and LC3B double-positive deposits, suggesting accumulation of DNA in autophagosomes or autolysosomes (Figure 2B). Incubation of cardiomyocytes with CCCP increased the levels of *Il6* and *Il1b* mRNAs (Figure 2C). E6446 had no effect on the number of PicoGreen and LC3B double-positive deposits, but it significantly reduced *Il6* and *Il1b* mRNA expression in CCCP-treated cardiomyocytes.

### In vivo administration of E6446

**Figure 3:**
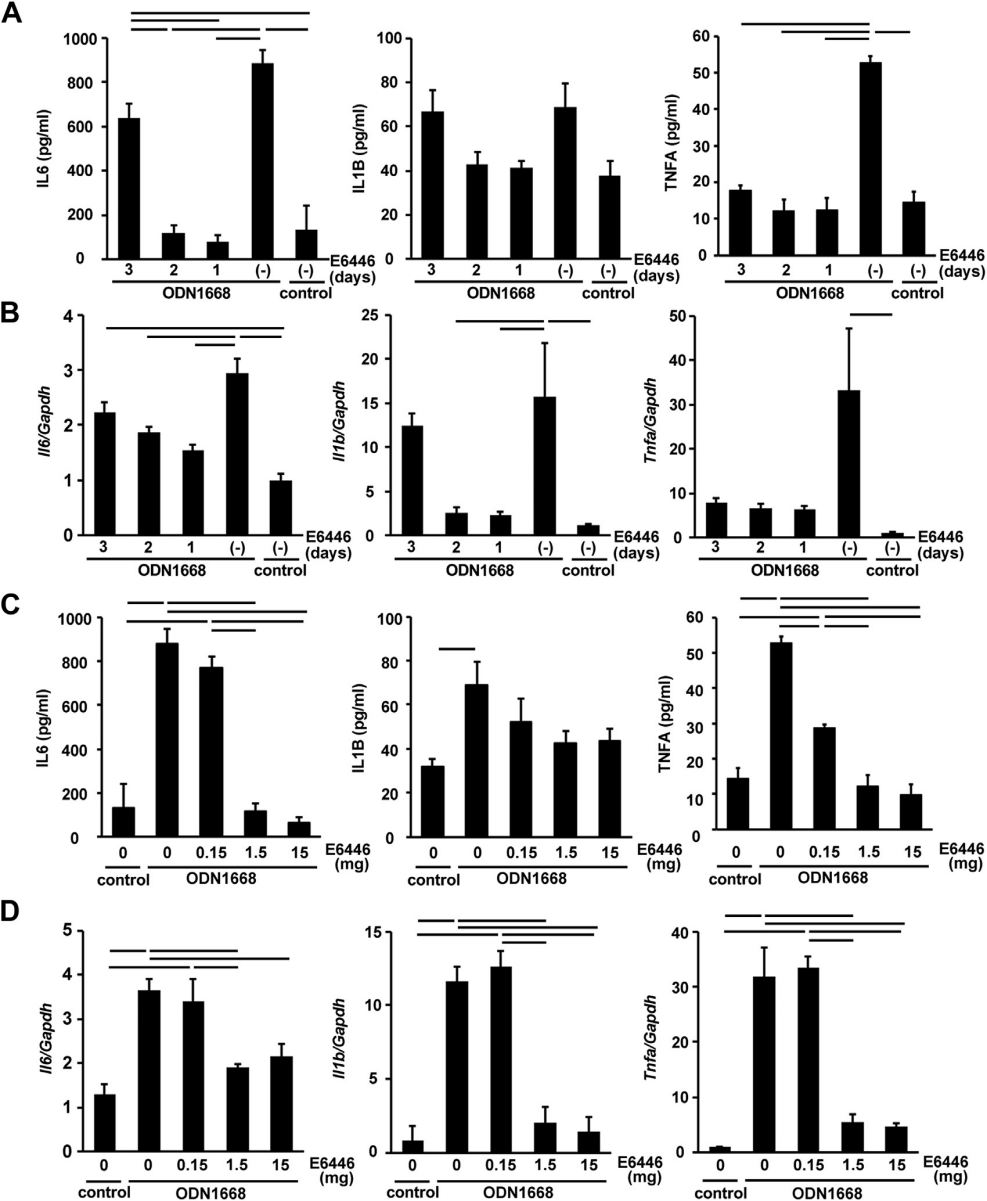
Determination of the Experimental Conditions for E6446 Administration The time dependence of **(A)** cytokine protein and **(B)** messenger ribonucleic acid (mRNA) expression in the heart after injection with ODN1668. Mice (body weight 24.4 to 25.8 g) were pretreated with oral administration of 1.5 mg/mouse (60 mg/kg) of E6446 1, 2, or 3 days before intraperitoneal injection with 60 μg/mouse of ODN1668. Two hours after ODN1668 injection, mice were sacrificed for analysis (see Figure 9A). Dose dependency in the inhibition of **(C)** cytokine protein and **(D)** mRNA expression after ODN1668 injection with increasing concentrations of E6446. Two days after oral administration with the indicated dose of E6446, mice were administered an intraperitoneal injection of 60 μg/mouse of ODN1668. Two hours later, mice were sacrificed for analysis (see Figure 9B). Data were normalized to the content of *Gapdh* mRNA in **B and D**. Values are mean ± SE (n = 3). Bars in graphs indicate p < 0.05. IL = interleukin; TNF = tumor necrosis factor; other abbreviation as in Figure 1.

To examine the in vivo efficacy of E6446 on inhibition of TLR9 and determine the experimental conditions for its administration in mice, 1.5 mg/mouse (60 mg/kg) of E6446 was orally administered to mice 3, 2, or 1 day before intraperitoneal injection of ODN1668. Two hours after the mice were administered ODN, the treated mice produced a higher level of IL-6 than control mice (Figure 3A). When E6446 was administered 2 or 1 day before ODN1668 injection, IL-6 levels were lower than the E6446-nontreated ODN1668-injected group and showed no significant difference compared with the control group without ODN1668 injection. When E6446 was administered 3 days before ODN1668 injection, the level of IL-6 did not differ from that in the nontreated group. To confirm the protein data, mRNA levels in the heart were measured (Figure 3B). The level of *Il6* mRNA in ODN1668-injected mouse hearts was higher than that in control hearts. When E6446 was administered 2 or 1 day before ODN1668 injection, *Il6* mRNA levels were lower than the E6446-nontreated ODN1668-injected group. When E6446 was administered 3 days before ODN1668 injection, the level of *Il6* mRNA differed from the control group but not from other ODN1668-injected groups. The level of *Il1b* mRNA in the E6446-nontreated ODN1668-injected mouse hearts was higher than that in the control hearts and showed a significant difference from that treated with E6446 1 and 2 days but not 3 days before ODN1668 injection; the levels of IL-1B protein were not significantly different among groups. The level of TNF-α in the ODN1668-injected mouse group was higher than that in the nontreated control group and in all the E6446-treated ODN1668-injected groups. The level of *Tnfa* mRNA in E6446-nontreated ODN1668-injected mouse hearts was higher than that in control hearts but showed no difference from all other groups. Thus, the inhibitory effect of E6446 on the induction of cytokines lasted over a period of 2 days, providing a rationale for every-other-day dosing.

The dose of E6446 necessary to inhibit the induction of cytokines was next examined. The serum level of IL-6 and TNF-α protein in mice treated with 1.5 or 15 mg/mouse of E6446 was lower than that in nontreated mice or in mice treated with 0.15 mg/mouse of E6446 but did not differ from that in control mice (Figure 3C). ODN1668 injection increased serum levels of IL-1B protein. E6446 administration produced no significant inhibitory effect on the increase of IL-1B protein. The levels of *Il6*, *Il1b,* and *Tnfa* mRNAs in hearts treated with 1.5 or 15 mg/mouse were significantly lower than those in the hearts of nontreated mice (Figure 3D). Thus, 1.5 mg/mouse of E6446 was administered every 2 days in the following experiments.

### Prevention of the development of heart failure by E6446

**Figure 4:**
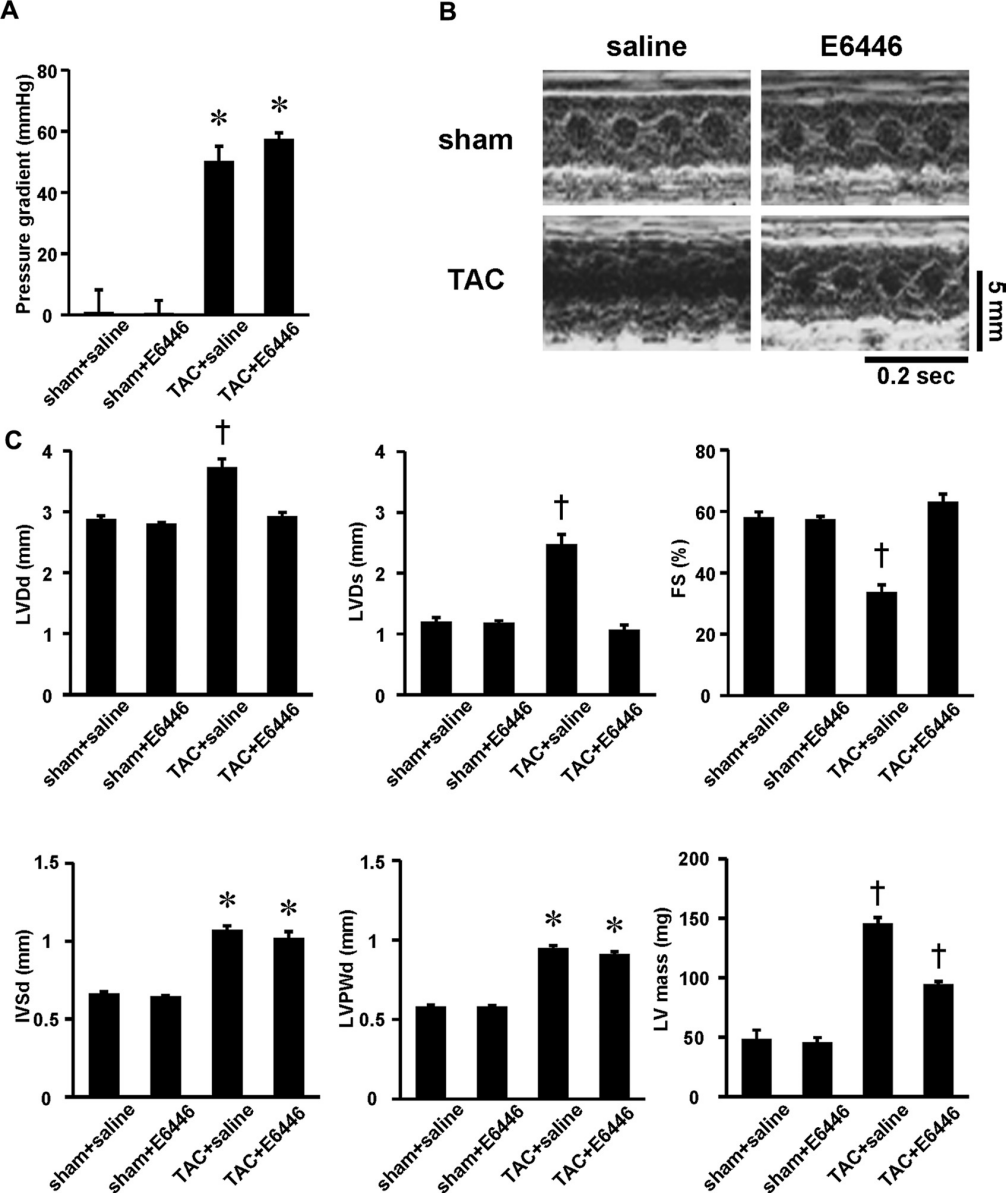
Improvement of Echocardiographic Parameters by Treatment With E6446 Initiated Before TAC The oral administration of E6446 (1.5 mg/mouse) was performed every 2 days from 2 days before transverse aortic constriction (TAC) (see Figure 10A). **(A)** Pressure gradient across TAC estimated by using a pressure monitor 1 week after operation. **(B)** Representative images of transthoracic M-mode echocardiographic tracing. Scale bars, 0.2 s and 5 mm, respectively. **(C)** Echocardiographic parameters of the mice treated with E6446 4 weeks after TAC (n = 4 in sham groups, n = 5 in TAC groups). Values are mean ± SE. *p < 0.05 versus sham-operated groups. †p < 0.05 versus all other groups. FC = fractional shortening; IVSd = end-diastolic interventricular septal wall thickness; LV = left ventricular; LVDd = end-diastolic left ventricular internal dimension; LVDs = end-systolic left ventricular internal dimension; LVPWd = end-diastolic left ventricular posterior wall thickness; other abbreviation as in Figure 1.

To investigate the efficacy of E6446 on the development of heart failure, mice orally received E6446 or saline 2 days before TAC and every 2 days for 4 weeks thereafter. There was no significant difference in pressure gradient across TAC between the E6446- and saline-treated groups 1 week after TAC (Figure 4A). Four weeks after TAC, saline-treated mice exhibited larger end-diastolic LV internal dimensions and end-systolic LV internal dimensions and lower fractional shortening than those in the sham-operated saline-treated group (Figures 4B and 4C). E6446 treatment significantly reduced LV chamber size and improved cardiac function in TAC-operated mice. TAC increased end-diastolic interventricular septal wall thickness (IVSd) and end-diastolic LV posterior wall thickness in saline- and E6446-treated mice. However, there were no significant differences in IVSd and end-diastolic LV posterior wall thickness between saline- and E6446-treated mice. LV mass was increased by TAC, and E6446 treatment attenuated the increase in LV mass in TAC-operated mice.

**Figure 5:**
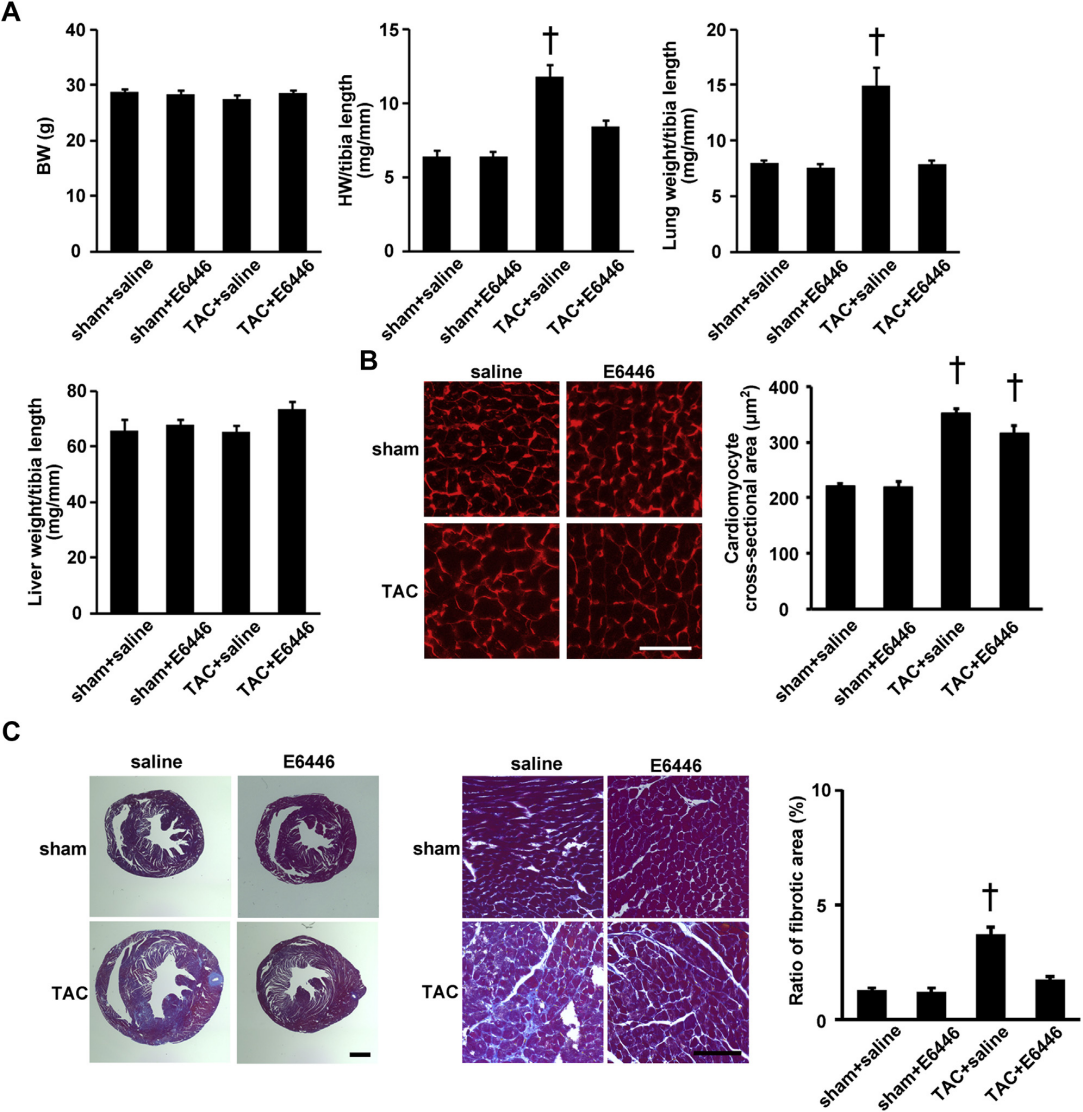
Improvement of Cardiac Phenotypes by Treatment With E6446 Initiated Before TAC The treatment with E6446 was performed every 2 days from 2 days before TAC. The mice were analyzed 4 weeks after the operation (see Figure 10A). **(A)** Physiological parameters of the mice treated with E6446 (n = 4 in sham groups, n = 5 in TAC groups). **(B)** The representative images of WGA-Alexa 555–stained heart sections. Scale bar, 50 μm. The **right graph** shows the cross-sectional area of cardiomyocytes. **(C)** Azan-Mallory–stained heart sections. **Left and middle panels** show the whole heart and magnified sections, respectively. Scale bars, 1 mm **(left panels)** and 100 μm **(middle panels)**. The **right graph** shows the ratio of the fibrotic area in the heart. n = 3 in sham-operated groups, n = 5 in TAC-operated saline-treated group, n = 4 in TAC-operated E6446-treated group in **B and C**. Values are mean ± SE. †p < 0.05 versus all other groups. BW = body weight; HW = heart weight; other abbreviations as in Figures 1 and 4.

**Figure 6:**
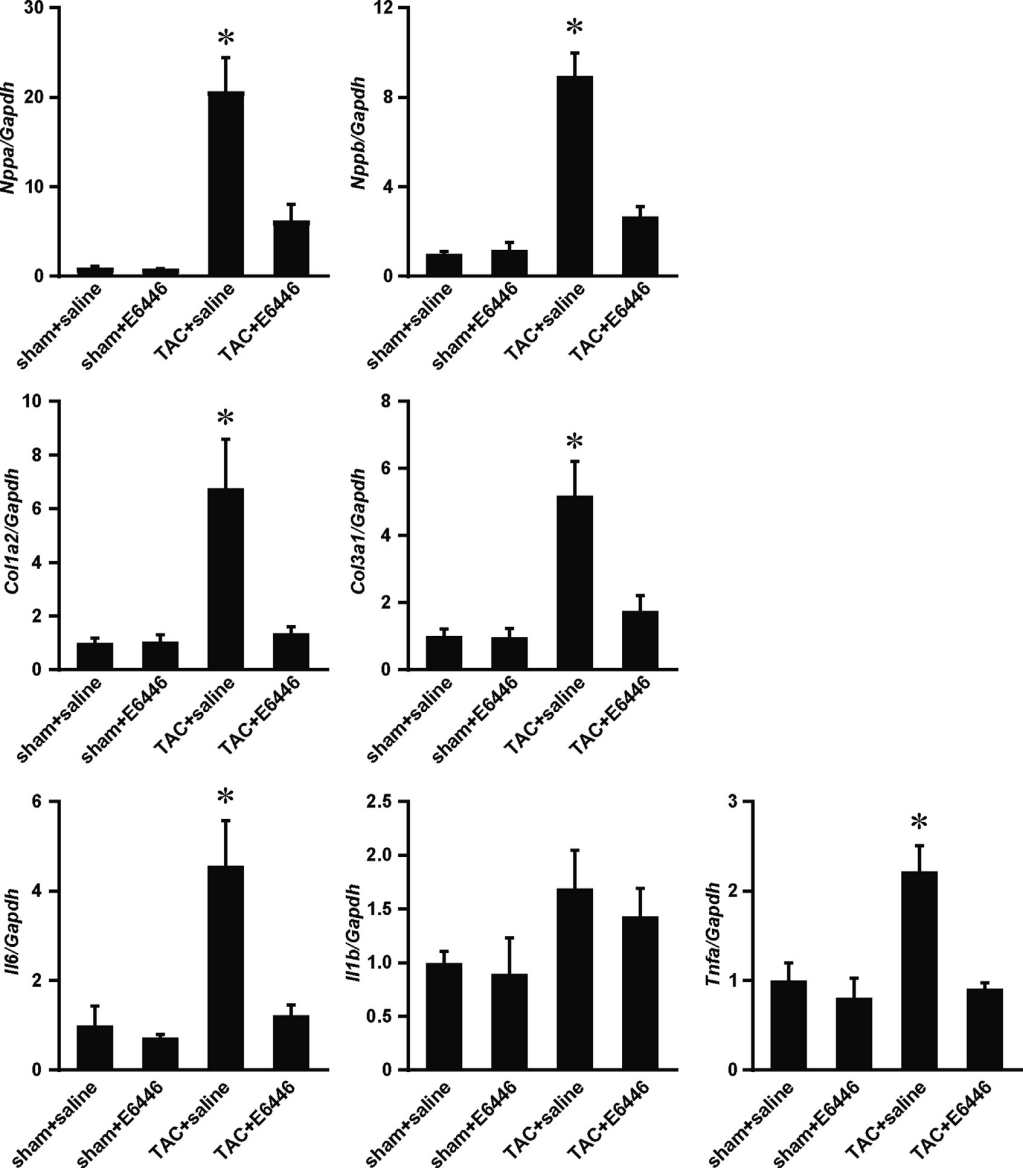
Effects of E6446 on mRNA Expressions The treatment with E6446 was performed every 2 days from 2 days before TAC. The mice were analyzed 4 weeks after the operation (see Figure 10A). Data were normalized to the content of *Gapdh* mRNA. Values are mean ± SE (n = 4 in sham groups, n = 5 in TAC groups). *p < 0.05 versus all other groups. Abbreviations as in Figures 1, 3, and 4.

There was no significant difference in body weight between the 4 groups (Figure 5A). TAC-operated saline-treated mice exhibited increases in the heart weight–to–tibia length ratio and the lung weight–to–tibia length ratio. E6446 significantly attenuated cardiac hypertrophy and lung congestion. TAC increased the cross-sectional area of cardiomyocytes in saline-treated mice, and the increase was significantly attenuated by E6446 treatment (Figure 5B). *Nppa* and *Nppb* mRNAs increased in TAC-operated saline-treated mice. E6446 attenuated the increases induced by TAC (Figure 6). TAC-operated saline-treated mice exhibited cardiac fibrosis, which was diminished in E6446-treated mice (Figure 5C). The mRNA levels of *Col1a2* and *Col3a1* increased in TAC-operated saline-treated mice. E6446 attenuated the increase in the mRNAs.

### Attenuation of inflammation in pressure-overloaded hearts by E6446

**Figure 7:**
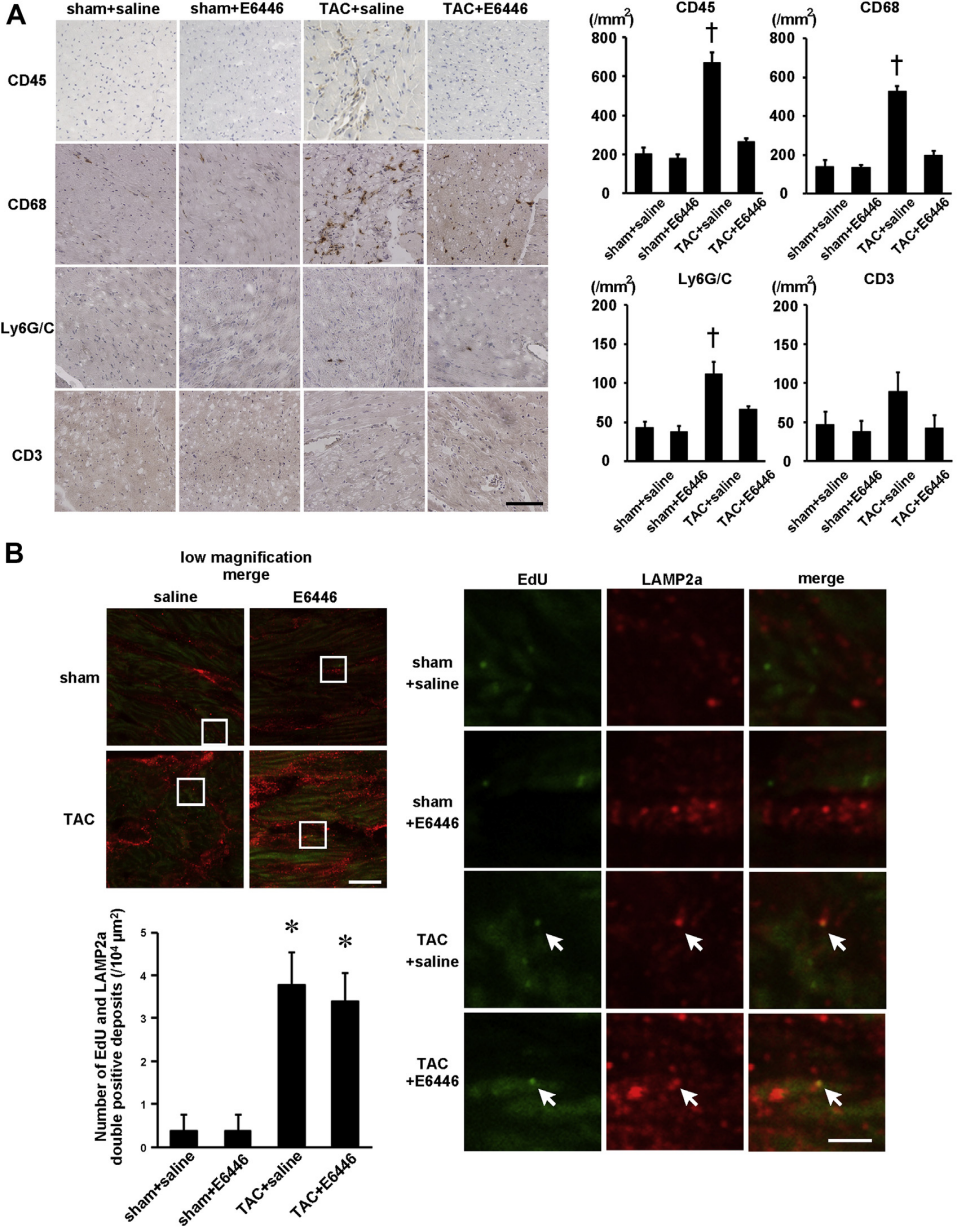
Attenuation of Inflammatory Responses With Treatment With E6446 Initiated Before TAC The treatment with E6446 was performed every 2 days from 2 days before TAC. The mice were analyzed 4 weeks after the operation (see Figure 10A). **(A)** Immunohistochemical analysis of the heart. Scale bar, 100 μm. The **right graphs** show the quantitative data for numbers of infiltrated inflammatory cells. **(B)** Deposition of mitochondrial deoxyribonucleic acid in lysosomes. Double staining of the heart sections with 5-ethynyl-2′-deoxyuridine (EdU) **(green)** and anti–lysosome-associated membrane protein (LAMP) 2a antibody **(red)**. Low-magnified images are shown in **left panels**. Scale bar, 10 μm. Higher magnified images of the squared areas are shown in **right panels**. Scale bar, 2 μm. **Arrows** indicate EdU and LAMP2a merged deposits. The **left graph** shows the number of EdU- and LAMP2a double-positive deposits. n = 3. Values are mean ± SE. *p < 0.05 versus sham-operated groups. †p < 0.05 versus all other groups. Abbreviations as in Figures 1 and 4.

TAC-operated saline-treated mice showed infiltration of CD45^+^ cells, including CD68^+^ macrophages in the heart, which was inhibited by treatment with E6446 (Figure 7A). Although increases in the mRNA expressions of *Il6* and *Tnfa* were detected in saline-treated TAC-operated mice, E6446 attenuated the induction of the mRNAs (Figure 6).

To label mitochondrial DNA, mice were injected with EdU 1 day before sacrifice. EdU specifically binds to mitochondrial DNA during active DNA synthesis in nondividing cardiomyocytes (7). LAMP2a is a marker for lysosomes. In TAC-operated saline- and E6446-treated hearts, EdU and LAMP2a double-positive deposits were observed (Figure 7B). There was no significant difference in the number of the double-positive deposits between TAC-operated saline- and E6446-treated hearts.

### Slowing the progression of heart failure by E6446

**Figure 8:**
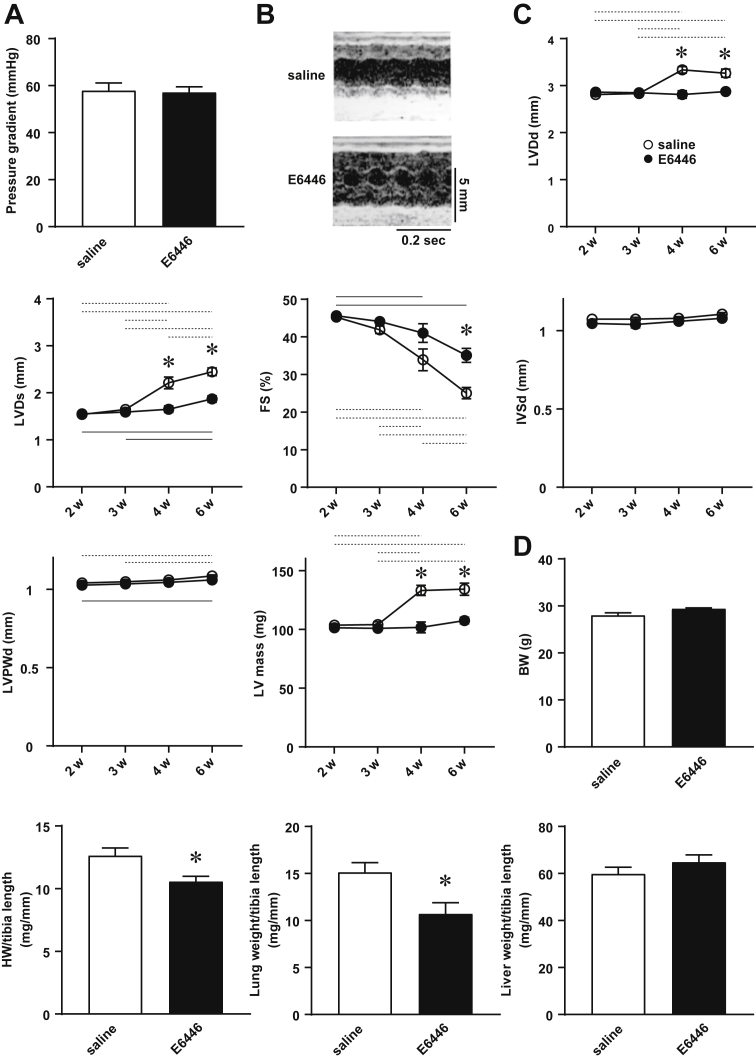
Improvement of Echocardiographic Parameters and Lung Congestion After Treatment With E6446 Initiated After TAC Two weeks after TAC, treatment with E6446 was performed every 2 days (see Figure 10B). Values are mean ± SE (n = 10). **(A)** Pressure gradient across TAC estimated by using a pressure monitor 1 week after operation. **(B)** Representative images of transthoracic M-mode echocardiographic tracing. Scale bars, 0.2 s and 5 mm, respectively. **(C)** Echocardiographic parameters. The parameters were examined for 6 weeks after TAC. **Open and closed circles** indicate saline-treated control groups and E6446-treated groups, respectively. The data were analyzed by using 2-way repeated measure analysis of variance followed by Tukey’s post hoc test. *p < 0.05 between the 2 groups at the corresponding time point. **Dotted lines** indicate p < 0.05 between the 2 saline-treated control groups at different time points. **Solid lines** indicate p < 0.05 between the 2 E6446-treated groups at different time points. **(D)** Physiological parameters 6 weeks after the operation. *p < 0.05 versus saline-treated control group. Abbreviations as in Figures 1, 4, and 5.

**Figure 9:**
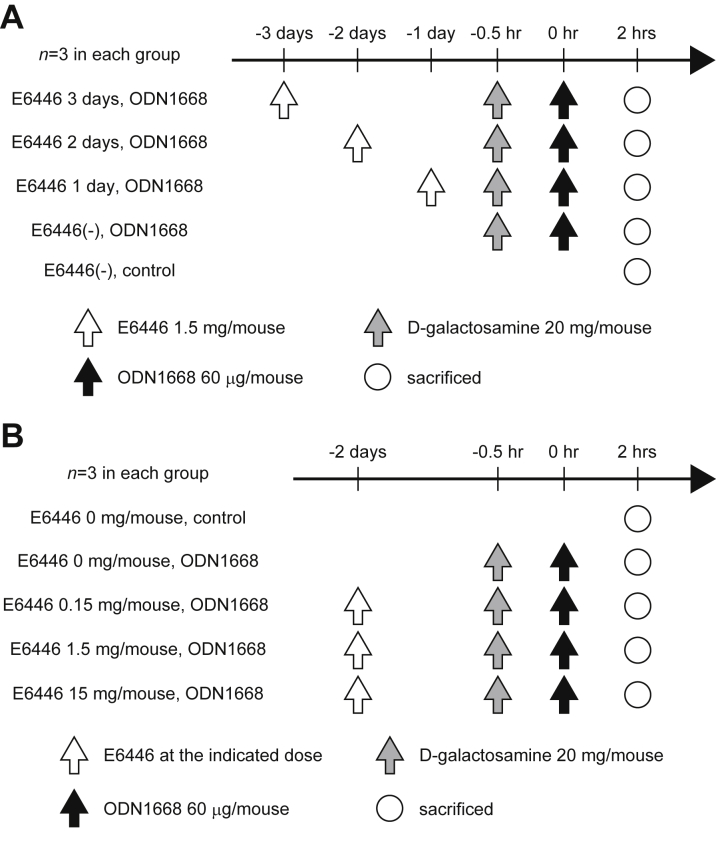
Schematic Protocol to Determine the Experimental Conditions for E6446 Administration **(A)** Schematic protocol to examine the time dependence of cytokine protein and mRNA expression in the heart after injection with ODN1668 (n = 3 for each group). Mice were pretreated with E6446 1.5 mg/mouse for 1, 2, or 4 days before injection of 60 μg/mouse of ODN1668. Two hours after ODN1668 injection, mice were sacrificed for analysis, following treatment with D-galactosamine. **(B)** Schematic protocol to examine dose dependency in the inhibition of cytokine expression after ODN1668 injection with increasing concentrations of E6446 (n = 3 for each group). Two days after administration with the indicated dose of E6446, mice received 60 μg/mouse of ODN1668, following D-galactosamine injection. Two h later, mice were sacrificed for analysis. Abbreviations as in Figures 1 and 3.

**Figure 10:**
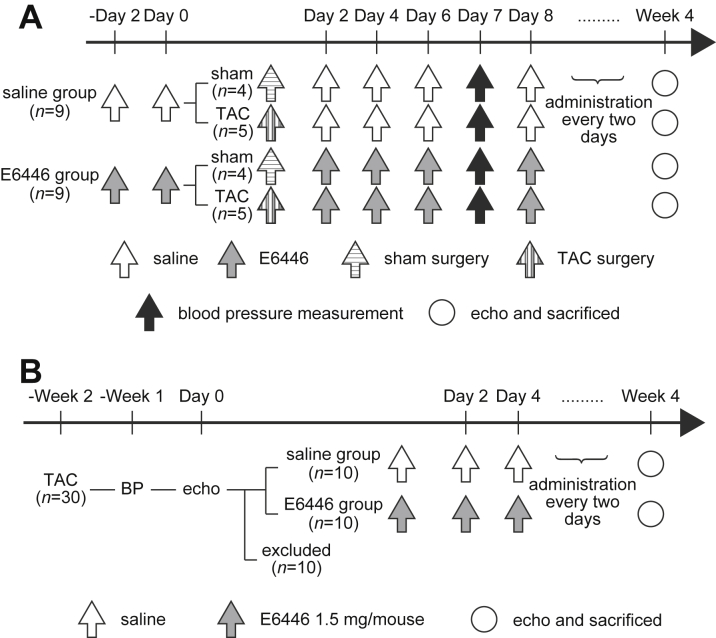
Schematic Protocols to Examine the Effect of E6446 on TAC-Induced Heart Failure **(A)** Schematic protocol to examine the effect of treatment with E6446 initiated before TAC on cardiac phenotypes. E6446 (1.5 mg/mouse) (n = 9) or saline (n = 9) was administered every 2 days from 2 days before TAC. Blood pressure was measured 7 days after TAC. Four weeks after TAC, mice were subjected to echocardiographic analysis and sacrificed. **(B)** Schematic protocol to examine the effect of treatment with E6446 initiated after TAC on cardiac phenotypes. Thirty mice were subjected to TAC operation for 2 weeks. Ten mice with fractional shortening >50% were excluded from the study. The remaining 20 mice were randomized to the saline- and E6446-treated groups and then administered saline or E6446 (1.5 mg/mouse) every 2 days. Six weeks after TAC, mice were subjected to echocardiographic analysis and sacrificed. Abbreviations as in Figures 1 and 4.

Finally, the effect of E6446 on the progression of an already established disease was examined. Mice were subjected to TAC operation (Figure 8A) and divided into 2 groups 2 weeks later. There were no significant differences in echocardiographic parameters between the 2 groups, which already exhibited chamber dilatation and cardiac dysfunction (Table 2). The mice were then administered E6446 or saline every 2 days for 4 weeks. LV chamber dilatation and cardiac dysfunction progressively worsened with time in both groups (Figures 8B and 8C). End-diastolic LV internal dimensions, end-systolic LV internal dimensions, and LV mass showed no significant difference between saline- and E6446-treated TAC-operated mice until 3 weeks after TAC. However, the parameters were significantly smaller in E6446-treated mice than those in saline-treated mice 4 and 6 weeks after the operation. Fractional shortening was significantly higher in E6446-treated mice than that in saline-treated mice 6 weeks after TAC. There were no significant differences in IVSd and end-diastolic LV posterior wall thickness between the 2 groups at any time point. The heart weight–to–tibia length and lung weight–to–tibia length ratios were significantly reduced by E6446 treatment (Figure 8D). Schematic protocols to examine the effect of treatment of E6446 on cardiac phenotypes are described in Figures 9 and 10.

**Table 2:** Echocardiographic Parameters of Mice Included in the E6646 Treatment Study 2 Weeks After TAC Operation

	Baseline		2 Weeks After TAC	
	Saline (n = 10)	E6446 (n = 10)	Saline (n = 10)	E6446 (n = 10)
LVDd, mm	2.30 ± 0.03	2.27 ± 0.03	2.81 ± 0.03∗	2.86 ± 0.03∗
LVDs, mm	0.88 ± 0.02	0.86 ± 0.02	1.54 ± 0.03∗	1.55 ± 0.02∗
LVFS, %	61.5 ± 0.71	62.3 ± 0.79	45.2 ± 0.77∗	45.6 ± 0.43∗
IVSd, mm	0.92 ± 0.01	0.92 ± 0.01	1.07 ± 0.01∗	1.05 ± 0.01∗
LVPWd, mm	0.86 ± 0.02	0.87 ± 0.01	1.04 ± 0.01∗	1.03 ± 0.01∗
Heart rate, beats/min	692 ± 3.9	695 ± 6.5	689 ± 7.2	672 ± 7.5
LV mass, mg	58.2 ± 1.4	58.9 ± 1.1	103.5 ± 3.1∗	101.4 ± 2.1∗

Values are mean ± SE. Thirty mice were subjected to TAC operation for 2 weeks. Ten mice with fractional shortening >50% were excluded from the study. The remaining 20 mice were randomized to the saline- and E6446-treated groups. The parameters of the mice were obtained 2 weeks after the operation by using echocardiography.

Abbreviations as in Table 1.

∗ p < 0.05 vs. corresponding control at baseline.

## Discussion

The present study showed that E6446 prevents the development of pressure overload–induced heart failure when administered before the cardiac event and also suppresses the progression of heart failure when started after cardiac dysfunction manifested. We have reported that TLR9 is essential in producing inflammatory cytokines in failing hearts (7). TLRs are essential in driving the recruitment of inflammatory cells and production of cytokines during cardiac remodeling (13). E6446 prevents cellular events activated by TLR9, exerting broader inhibitory effects on inflammatory cytokine production, and thus the treatment of heart failure with the inhibitor has an advantage over the therapy neutralizing only 1 cytokine. The near-complete rescue of TAC-induced LV dilatation and dysfunction by E6446 pretreatment suggests that the TLR9-signaling pathway is the dominant pathway for inducing adverse ventricular remodeling, with a limited role for other pathways such as nucleotide-binding domain leucine-rich repeat containing protein 3 (NLRP3) and cyclic GMP-AMP synthase (cGAS)-stimulator of interferon genes (STING) activation by mitochondrial DNA released in the cytosol in the setting of mitophagy dysfunction (14).

In human peripheral blood mononuclear cells or mouse spleen cells, E6446 diminished IL-6 production in response to CpG ODN (8). A 100-fold higher concentration of E6446 inhibited cytokine production in response to the imidazoquinoline compound R848, which is a TLR7/8 agonist (15). When C57BL/6 mice were orally treated with E6446, E6446 completely inhibited CpG ODN–induced IL-6 production in sera but not R848- and LPS-mediated cytokine production (8). Consistent with these results, our findings indicate that E6446 specifically inhibited the expression of inflammatory cytokines through a TLR9-dependent pathway but not TLR4- or TLR7-dependent pathways in adult cardiomyocytes. Thus, E6446 has high specificity to TLR9. In mouse bone marrow–derived dendritic cells, E6446 potently inhibited IL-6 production induced by CpG ODN but not by TLR3 ligands (9). However, E6446 was a potent inhibitor of IL-6 induction by single-stranded RNA, a TLR7/8 agonist, but a relatively poor inhibitor of IL-6 induction by R848, suggesting that the ability of E6446 to suppress TLR7/8 might be ligand dependent. Based on our data showing the importance of TLR9 signaling in the development of inflammation and heart failure and its specificity to TLR9 in cardiomyocytes, the cardioprotective action of E6446 is TLR9 mediated. However, we cannot exclude the possibility that TLR7/8 is involved in the effect of E6446 on the development of heart failure.

E6446 inhibits in vitro DNA−TLR9 interaction via an association with DNA but not with TLR9 (9). Furthermore, E6446 accumulates in the intracellular acidic compartment. Mitochondrial DNA is accumulated in autolysosome and coexists with TLR9 in failing hearts (7). DNase II activity was up-regulated in hypertrophied hearts but not in failing hearts. The incomplete digestion of mitochondrial DNA would be due to the loss of up-regulation of DNase II activity. Mitophagy impairment occurs in the TAC-induced mouse heart failure model (16). Thus, it is also possible that impairment of mitophagy or lysosomal permeabilization or lysosomal dysfunction might result in the accumulation of mitochondrial DNA in autolysosome. Our data in this study showed that there was no significant difference in the number of EdU and LAMP2a double-positive deposits between TAC-operated saline- and E6446-treated hearts. This outcome suggests that E6446 has no effect on mitochondrial DNA accumulation in autolysosomes. Thus, we can assume that the E6446 administered accumulates in lysosomes in the cardiomyocytes and interacts with mitochondrial DNA. When E6446 was orally administered to mice before TAC, E6446 inhibited TLR9 signaling by interfering with the mitochondrial DNA−TLR9 interaction and subsequent development of inflammation and heart failure.

We showed that *Tlr9*^–/–^ mice are more resistant to pressure overload than control mice, and inhibitory ODN to TLR9 (ODN2088) improved survival in TAC-operated wild-type mice when administered before TAC (7). However, administration of a drug before cardiac events is not clinically relevant. The results indicate that E6446 can slow the development of heart failure even after cardiac dysfunction manifested. Thus, E6446 or other immunomodulatory therapy can be used to prevent or delay pressure overload–induced heart failure.

### Study limitations

This study shows the therapeutic effects of a TLR9 inhibitor, E6446, on mouse pressure-overload heart failure model. Obviously, further studies are necessary to translate the findings into human heart failure therapy. TAC-induced mouse model does not fully represent the complex features of clinical heart failure. To establish the clinical feasibility of E6446 treatment for heart failure, the effects of E6446 on different heart failure models have to be examined, such as myocardial infarction. In addition, we used young healthy mice in this study. However, in most patients, and particularly in elderly patients, heart failure is accompanied by a range of comorbidities, such as hypertension, diabetes mellitus, renal dysfunction and hyperlipidemia. Such factors may influence on the cardioprotective effect of E6446 in heart failure. Thus, further investigation using various disease models is required to clarify the clinical target of E6446 treatment. Furthermore, it will be important to validate the findings in large animal models and ultimately in human patients. It remains unclear whether mitochondrial DNA is accumulated in autolysosomes and mitochondrial DNA-TLR9 axis is involved in the genesis of inflammation in human failing hearts.

## Conclusions

Heart failure is the result of various cardiac diseases such as myocardial infarction, high blood pressure, cardiomyopathy, valvular diseases, arrhythmia, and congenital heart diseases. Elevated levels of inflammatory mediators have been identified in patients with heart failure, including heart failure with reduced and preserved ejection fraction, as well as short-term decompensated heart failure (1). Thus, investigation of the involvement of the TLR9-signaling pathway in other mouse or larger animal heart failure models and various types of human heart failure is warranted. We ultimately will be able to identify subsets of patients with heart failure who will benefit from inhibition of TLR9 signaling.

Perspectives **COMPETENCY IN MEDICAL KNOWLEDGE:** Heart failure is a major health threat in the developed countries with high morbidity and mortality. Novel and effective therapeutic agents against heart failure need to be developed. Inflammation and proinflammatory cytokines play an important role in the pathogenesis of heart failure. Inflammatory mediators can be therapeutic targets in heart failure.

**TRANSLATIONAL OUTLOOK:** A TLR9 inhibitor, E6446, exerted a beneficial effect on attenuating the development or progression of heart failure in a pressure overload–induced mouse model. E6446 treatment has an advantage over targeted anticytokine approaches using biological response modifiers, because it modulates a broad spectrum of inflammatory mediators. Thus, it may be a new promising therapeutic agent for human heart failure.
